# Fabrication and application of indium-tin-oxide nanowire networks by polystyrene-assisted growth

**DOI:** 10.1038/s41598-017-01385-0

**Published:** 2017-05-09

**Authors:** Qiang Li, Feng Yun, Yufeng Li, Wen Ding, Ye Zhang

**Affiliations:** 1 Key Laboratory of Physical Electronics and Devices for Ministry of Education and Shaanxi Provincial Key Laboratory of Photonics & Information Technology, Xi’an Jiaotong University, Xi’an, Shaanxi P.R. China; 2 Solid-State Lighting Engineering Research Center, Xi’an Jiaotong University, Xi’an, Shaanxi P.R. China

## Abstract

The fabrication and application of polystyrene (PS)-assisted ITO nanowire (NW) networks are reported. The ITO-NW networks are fabricated by means of electron-beam deposition via PS. This method has the advantages of low-temperature (~300 °C), low-cost, facile and efficient operation. The growth mechanism of PS-assisted ITO NWs was analyzed in detail, and the morphology of which could be regulated by the size of PS. X-ray diffraction and high-resolution transmission electron microscope show that the ITO NWs are close to an integral cubic lattice. The transmittance of ITO-NW networks layer is above 90% after 400 nm and the sheet resistance is ~200 Ω/□. When they applied on vertical blue and green LEDs, the light output power all has been improved ~30%. And, the resistive switching behaviors of ITO-NWs were measured and analyzed in Ag/ITO-NW networks/Al capacitor. The application of ITO-NW networks on special morphological devices was discussed. The PS-assisted ITO-NW networks show a strong researching and application value.

## Introduction

Transparent conductive film (TCF) is a kind of thin film which can conduct electricity and have high transparency in the visible range. It’s mainly used in the window material of photoelectric devices, such as light-emitting diodes (LED)^1^, solar cells^2, 3^, flat panel displays^4^, etc. Indium-tin-oxide (ITO) and doped zinc oxide (ZnO) are the most widely used and studied in recent years. ITO film is dominant in the market because of its mature technology^5^. Nowadays, doped-ZnO materials (such as ZnO doped with Aluminum (AZO)^6^, Gallium (GZO)^7–10^, Indium (IZO)^11^, Magnesium^12, 13^, and gold^14, 15^, etc.) have been researched deeply, and made some breakthroughs in the application of flexible devices^16–18^. However, due to their limitations of the preparation process and the stability of the resistivity, the main material of TCF is still ITO at this stage. Due to the high surface-volume ratio and excellent photoelectric properties of network based on nanomaterials, the nanoscal structures (e.g., nanorods, nanowires, nanotetrapods, and nanobelts) of semiconducting metal oxides have attracted much attention^19, 20^. So, ITO nanowires (NWs) have been fabricated and researched due to the expected improvement in the performance of various device applications over planar ITO thin film based devices^21–30^.

Up to date, two mechanisms have been discussed in ITO nanowires growth, namely Au-assisted vapor-liquid-solid (VLS) growth^31–33^ and self-catalytic VLS growth^34–36^. In Au-assisted VLS growth of ITO NWs, the growth temperature is above 800 °C and the complete removal of catalyst Au particles poses a challenge to obtain high purity ITO NWs. In self-catalytic VLS growth, however, the material to be grown as nanowires itself acts as a catalyst for nanowire growth. The growth temperature is much lower (below 400 °C), but the main drawback still remains in the effective control over the diameter of the nanowires. At the same time, the lateral conductive performance of ITO-NWs layer is very week, because a dense ITO-NW network is difficult to form base on the above two mechanisms, which seriously restricts the application of ITO nanowires. Therefore, we need new ways to address these issues.

**Figure 1:**
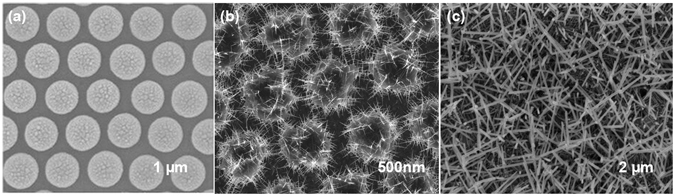
(**a**) PS assembly on wafer, (**b**) ITO NWs deposited on samples, (**c**) PS lift-off and ITO NW networks fabricated.

**Figure 2:**
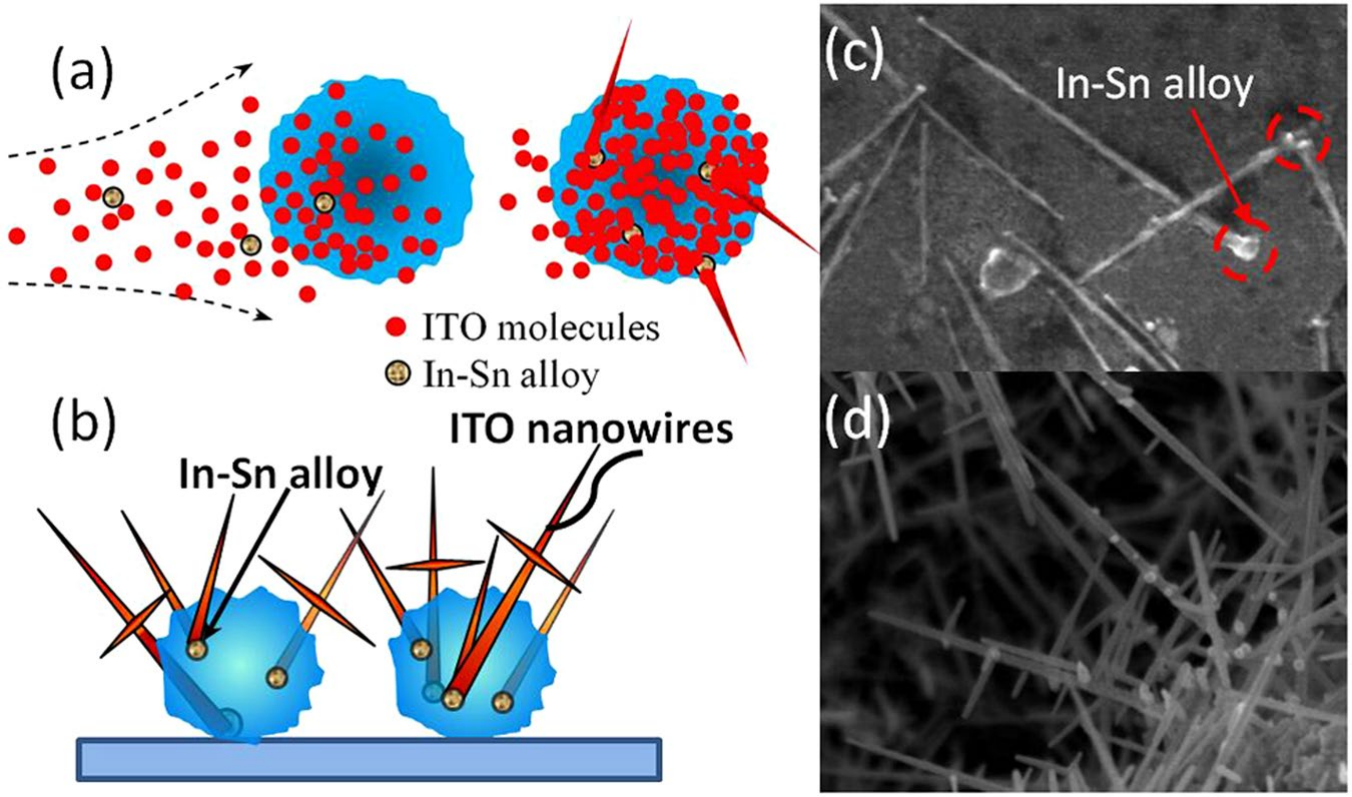
(**a**) The schematic diagram of ITO-NWs growth process; (**b**) The internal morphology of the PS after growing the ITO-NWs; (**c**) A single complete nanowire; (**d**) the branched structure of ITO-NWs.

In this study, the fabrication and application of ITO-NW networks are reported. The ITO-NW networks are fabricated by means of electron-beam deposition via polystyrene (PS). This method has the advantages of low-temperature, low-cost, facile and efficient operation^37^, and the PS spheres are prepared on substrate by self-assembly, following by the use of electron beam evaporation (EBE) to fabricate ITO NWs via PS sites. PS were then removed by annealing to obtain ITO NWs with uniform distribution and better crystal quality. Meanwhile, the morphology of the NWs became the interwoven networks. The reason is that all the residual PS becomes liquid at a high temperature, the liquid carrying the NWs flows on the surface of substrate freely. When the PS was decomposed completely, a uniform interwoven network of NWs was left and adhered to the substrate. Figure 1 shows the images of scanning electron microscope (SEM) on each stage. ITO NWs were grown on the In-Sn particles via molten PS spheres (Fig. 2a), and the In-Sn alloys were wrapped inside the PS (Fig. 2b). Figure 2c shows the morphology of a whole ITO-NW which was taken out completely from PS spheres without any damage. Obviously, there is a ball at the root, which is the nucleation point of the ITO-NW growth. Figure 2d is the partial image of the interwoven networks, showing that the nanowires have the branched structure. In addition, the ITO nano-tree can be prepared in a directed way by this method (see Supplementary).

The photoelectric properties of ITO-NW networks were measured, and the morphology of which could be regulated by the PS size. ITO-NW networks prepared by this method were applied on the vertical blue/green LEDs, and the light extraction efficiency (LEE) has been further improved. At the same time, the resistive switching behaviors of ITO-NW networks were observed by using Ag/ITO-NW networks/Al sandwich structure. The reversible and steady bistable resistive switching behaviors provided the potential for nonvolatile memory application. The ITO-NW networks can also be used on some devices with special morphologies (such as pyramids, holes, etc.), which further extends the application.

## Results

### Properties of ITO-NW networks

**Figure 3:**
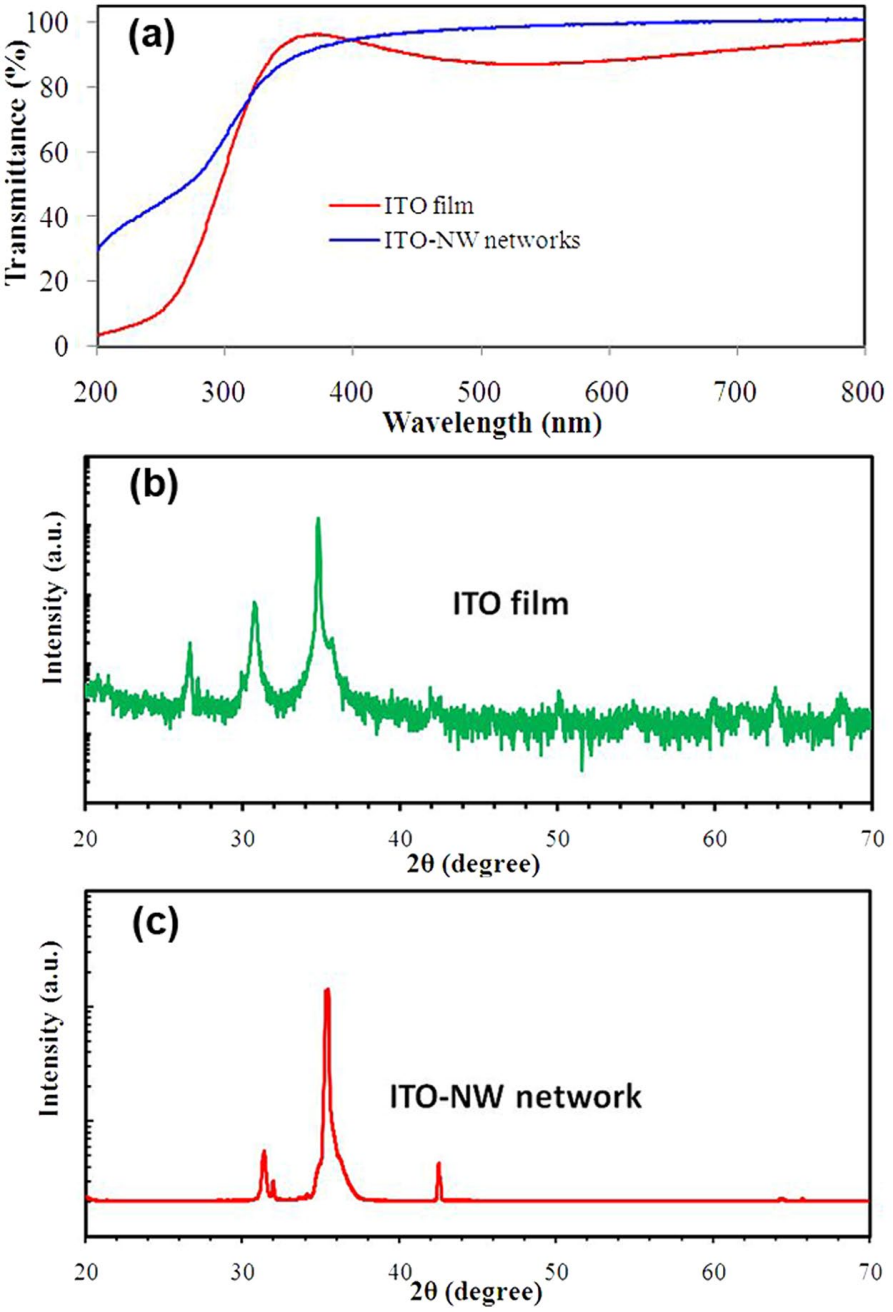
(**a**) The transmittance of ITO film and ITO-NW networks. The XRD spectrum of (**b**) ITO film and (**c**) ITO-NW networks.

The optical transmittance and sheet resistance of the ITO-NW networks were measured. The results are shown in Fig. 3. The ITO-NW networks were prepared by PS spheres of 670 nm for 20 min at 300 °C. ITO film and ITO-NW networks were both fabricated on quartz substrate, and then annealed for 5 min at 300 °C under the nitrogen ambient. After the wavelength of 400 nm, the transmittance of ITO-NW networks is significantly higher than that of ITO bulk film. The pores in the NW networks are much larger than that in dense film, and the light is easier to pass through. The surface with ITO-NW networks has characteristics of low reflectivity and large absorption because of light trapping^38–40^, but the transmittance of which still has been improved. Whereafter, the sheet resistance was measured, the ~16 Ω/□ and ~200 Ω/□ for ITO film and ITO-NW networks, respectively. The sheet resistance of the bulk material is smaller than that of ITO-NW networks, because there are more air gaps and oxygen vacancies in networks.

X-ray diffraction (XRD) was measured to verify the growth quality of ITO NWs. From Fig. 3b,c, the XRD profile for the ITO NWs film on the quartz show diffraction peaks corresponding to the cubic structure^41^. The peaks at 2*θ = *30.5 and 35.4°, corresponding the two major (222) and (400) peaks of the ITO NWs film, are very obvious, which suggest that the ITO NWs are closer to an integral cubic lattice.

### Morphology of ITO NW networks

**Figure 4:**
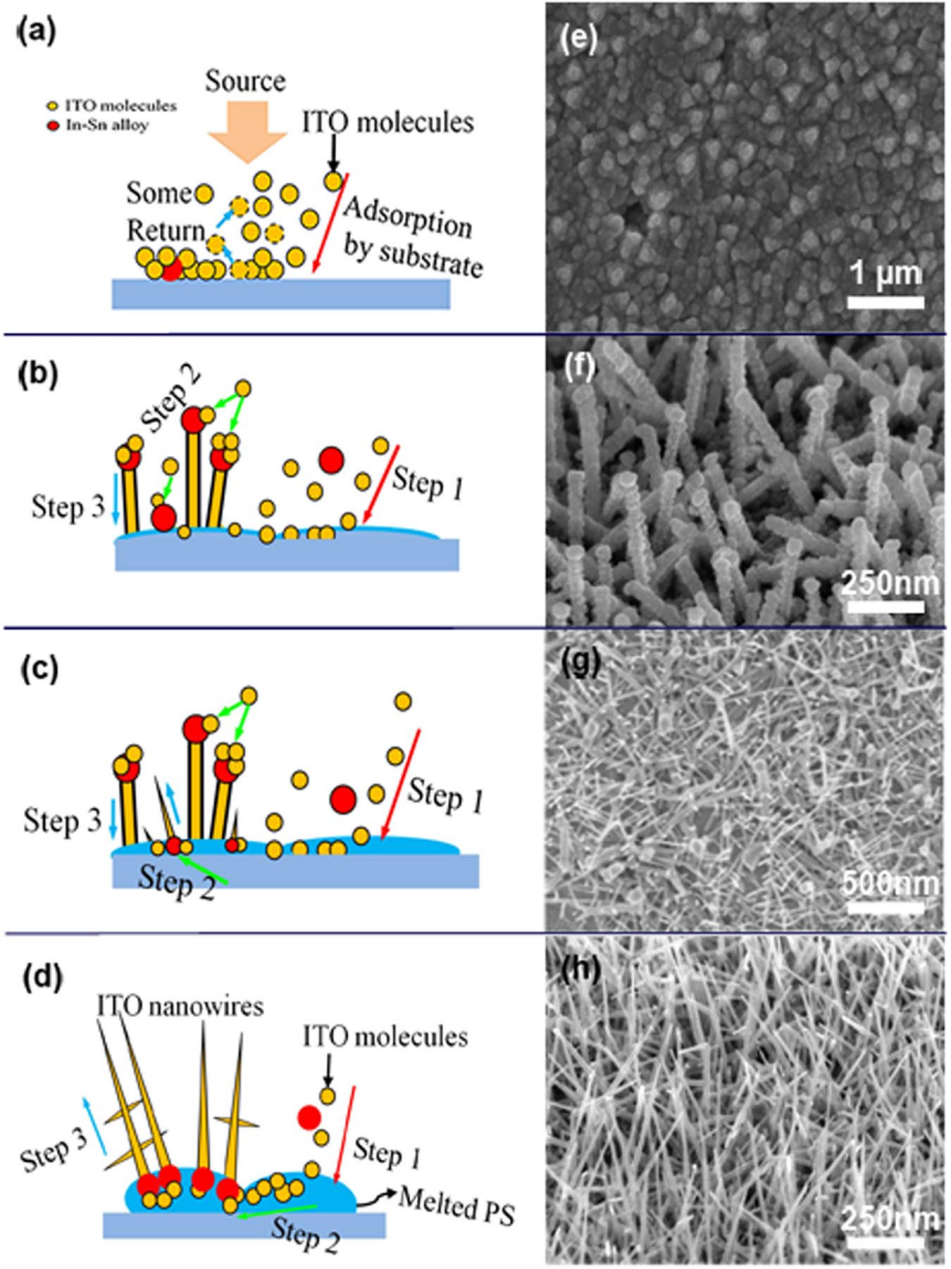
The growth schematic diagrams and SEM images of PS-catalytic ITO NWs. (**a**) The substrate without PS, most of ITO molecules were adsorbed and get together on the substrate, while another portion of the molecules was bounced back; (**b**) the substrate with small PS spheres, (**c**) the substrate with a certain size of PS spheres, (**d**) the substrate with large PS spheres. In (**b–d**), step1: ITO molecules were adsorbed by the molten PS; step 2: some molecules be adsorbed directly on the surface of In-Sn alloy; step 3: the ITO NWs begin to grow. The SEM images of deposited ITO on GaN substrate (**e**) without PS, and with (**f**) 200 nm, (**g**) 500 nm, (**h**) 670 nm diameter of PS spheres.

Four groups of samples were prepared, namely GaN substrate without PS, and with 200 nm, 500 nm, 670 nm dia. of PS spheres. Figure 4 illustrates the growth schematics and also the SEM images of the morphology control of ITO NWs by using different diameters of PS spheres.

Since the EBE deposition of ITO is a dynamic gas and solid phase transformation process, the vaporized ITO molecules reaching the substrate surface will be either adsorbed or coagulated. Some indium-tin(In-Sn) alloy liquid droplets have formed, when the temperature is above 160 °C. (In-Sn eutectic melting point is generally in the 120–160 °C^42^). If no PS spheres were present on the substrate, the surface adsorption capacity of substrate would be relatively small. Most of the hybrid molecules (In_2_O_3_, SnO_2_) would be adsorbed and grown on the entire substrate, while a small portion would be bounced back, as shown in Fig. 4a and substantiated by the SEM morphology in Fig. 4e. Under such conditions, the In-Sn alloy droplets were covered by the particles of hybrid molecules to form a dense film on the substrate, instead of growing along any fixed crystal orientation and forming NWs.

When the substrate is coated with PS spheres, however, the PS is in the molten state under the temperature of 300 °C, and the surface adsorption energy is larger. In this case, the ITO molecules and In-Sn alloy are adsorbed preferentially by the melted PS spheres. In-Sn alloy keeps its liquid sate in PS, and more impinging atoms will be absorded because of the higher sticking coefficient. Nucleation occurs at the droplet/PS interface, resulting in the ITO NWs growth. If the PS diameter is too small, the adsorption capability is not strong enough to bind a large number of alloy particles. Those In-Sn particles get rid of the shackles of PS with the growth of NWs, and which are grown on the top of NWs. When the In-Sn droplets escaped from PS, some molecules could be adsorbed directly on the surface of In-Sn alloy. Therefore, the surface of ITO NWs is rough and diameter is large in this mode, forming ITO nanorods, as shown in Fig. 4b and exhibits SEM morphology as shown in Fig. 4f.

The bigger the diameter of PS is, the stronger the adsorption capability becomes. At a certain dimension as Fig. 4c, only some large In-Sn particles can escape to form nanorods from top to down as Fig. 4b. Most of the small In-Sn alloy-droplets are wrapped within the molten PS to allow NWs grow from down to top with needle shape. This is observed in the mixed morphology of Fig. 4g.

When the PS is large enough, all the In-Sn alloys are bounded by PS. The ITO molecules are absorded by PS firstly, and then enter into the In-Sn droplets. Nucleation occurs preferentially at the point with the lowest interfacial energy, resulting in the predominance of needle-shaped nanowire growth as shown in Fig. 4d,h.

A close observation of Fig. 4f–h reveals the different growth mode. In Fig. 4f, the top of every NW has a ball, and the NWs have large diameter and rough surface. Figure 4g, however, bears two kinds of NWs, large diameter and rough surface with ball head, and small diameter with needle shape. Figure 4h represents the third growth mode with clean NWs of small diameter and needle shape. Therefore, when using PS as assisted material, tuning the size of PS is an effective way to regulate the morphology of ITO NW networks.

**Figure 5:**
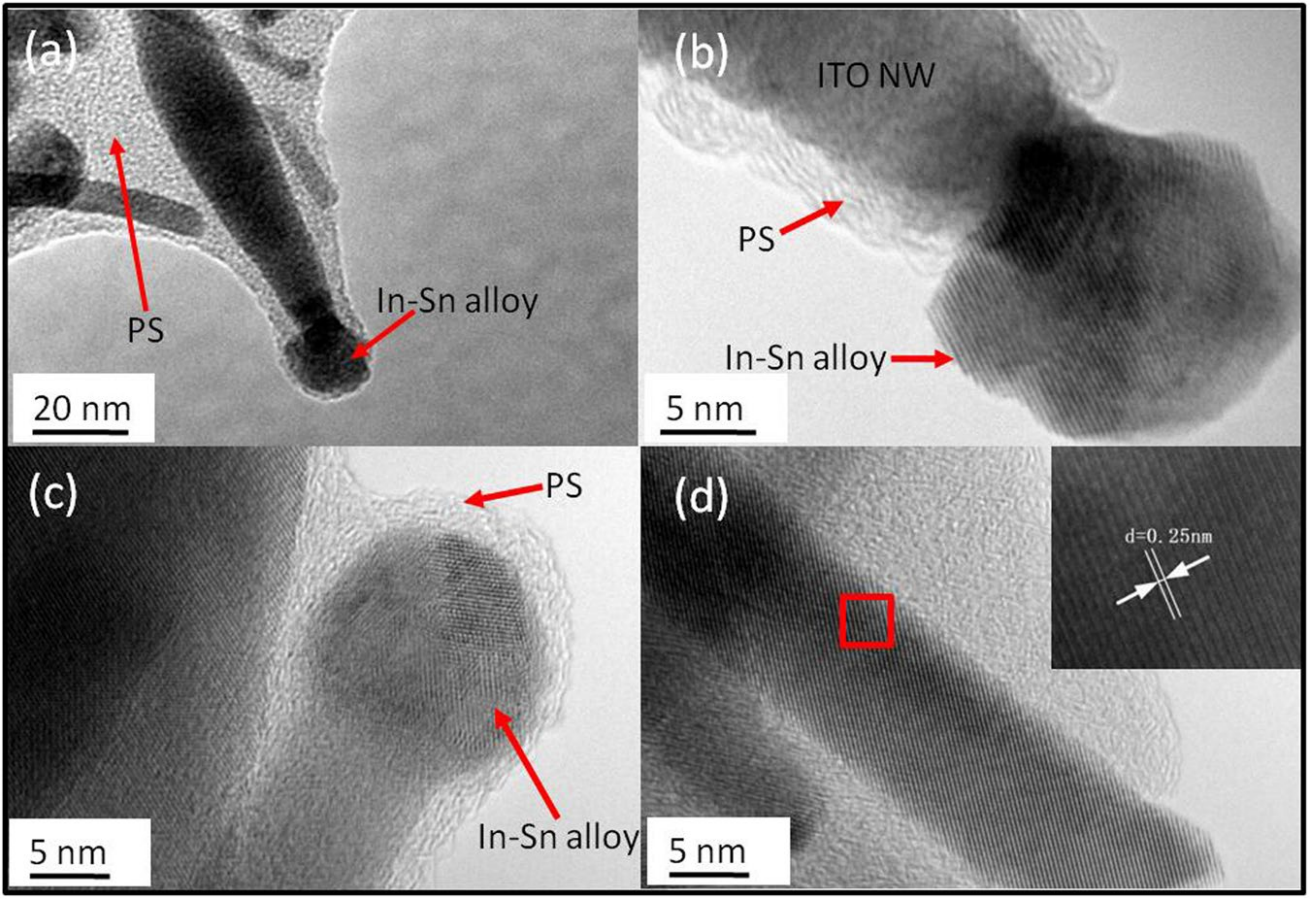
(**a**) The TEM image of ITO NWs at the beginning stage of growth; The HRTEM images of (**b**) the upper part of ITO nanorods; (**c**) the root part of needle-shaped NWs; (**c**) the trunk of NWs.

The transmission electron microscope (TEM) was used to characterize the structure of the fabricated NWs. At the initial stage of NWs growth, the In-Sn alloy and the nanowires were coated by PS. from Fig. 5a, the alloy particles were trying to get rid of the shackles of PS by the continuous growth of NWs. Figure 5b,d show the high resolution TEM (HRTEM) images of the In-Sn alloys (at the top/root of nanowires) and the trunk of NWs. The ball particles of In-Sn alloys were polycrystal in Fig. 5b,c.When the propelling force from NWs growth was greater than the binding force of PS, the alloy particles could escape from the PS (Fig. 5b), otherwise the ones were wrapped in PS (Fig. 5c). The Fig. 5d clearly showed the high degree of crystallinity for a complete ITO nanowire. The spacing between lattice fringes is 0.25 nm, which was well coincided with the “d” spacing of the cubic phase of In_2_O_3_^41^.

### ITO NW networks applied on vertical LED

**Figure 6:**
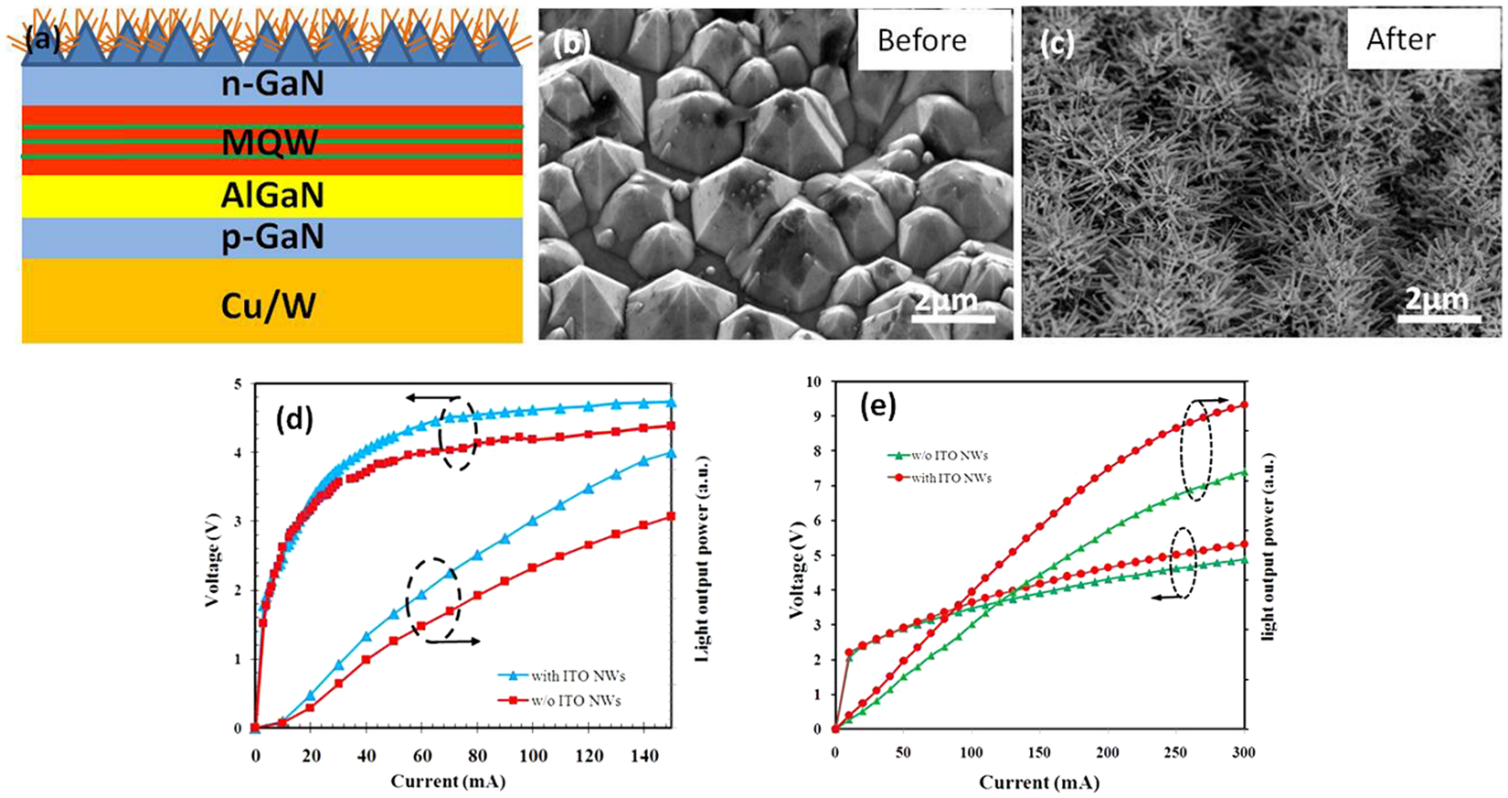
(**a**) The diagram of ITO-NW networks on vertical LED structure. (**b**) The surface of n-GaN was etched by KOH solution, showing the morphology of pyramid. (**c**) The SEM image of ITO-NW networks on pyramids. The I-V and light output power of (**d**) blue LED and (**e**) green LED.

The ITO-NW networks are fabricated on the vertical blue (λ = 450 nm) and green (λ = 519 nm) LED devices, respectively. The results are shown in Fig. 6.

First, two conventional LED structures are grown on sapphire by using metal-organic chemical vapor deposition (MOCVD). The blue-LED structure consists of five periods of InGaN/GaN quantum wells (QWs, 5 nm well, 12 nm barrier) on a 2 µm n-type GaN:Si (n-GaN) layer, designed for an emission wavelength at around 450 nm. And the green one consists of nine periods of InGaN/GaN QWs (3 nm well, 9 nm barrier) on a 3 μm n-type GaN:Si (n-GaN) layer, the emission wavelength at ~519 nm. AlGaN is the electron blocking layer (EBL, 20 nm) for both structures. The p-GaN layer is 300 nm for blue LED, and 120 nm for green one. Ni/Ag/Ti/Au layer is used as p-contact. After removing the sapphire substrate by laser lift-off process, the undoped-GaN is etched away using inductive coupled plasma (ICP)^43^. The hexagonal pyramid structures on the n-GaN layer (Fig. 6b) were obtained by dipping the sample in 6 mol/L KOH solution at 60 °C for 15 min. For the reference-LED (R-LED), the n-contact metal layer (Al/Ti/Au) is deposited on n-GaN directly. For the LED with ITO-NW networks (NW-LED), the standard optical lithography is used to cover the n-contact layer by photoresist before ITO NWs growth. Finally, ITO NWs are fabricated via PS spheres (~670 nm).

The electrical and optical properties of the vertical-LED (VLED) with and without ITO-NW networks are evaluated. Figure 6d,e show the I-V and light output power of VLEDs. For blue LEDs, the operation voltage @150 mA is 4.4 V for R-VLED (w/o ITO NWs), and 4.7 V for NW-VLED (with ITO NWs). The two values are very close, indicating that ITO NWs have little effect on the electrical properties. Moreover, the relative light output power of the NW-VLED increased by 31% comparing with that of R-VLED @150 mA. For green LED, the operation voltages of R-VLED and NW-VLED are 4.88 V, 5.31 V @300 mA, respectively. The relative light output power of the NW-VLED shows about 26% enhancement comparing with that of R-VLED @300 mA.

The ITO-NW networks have been reported as a light waveguide because of surface scattering effect^44, 45^. The light extraction efficiency (LEE) would be enhanced by limiting more photons in the escape cone. And, the ITO-NW networks film has the characteristics of gradient refractive index because of the different volume ratios of air and ITO material. Both of the transmittance and the critical angle have been improved by ITO-NW networks. So, the light output power of the NW-VLEDs was enhanced significantly.

### Resistive switching behaviors of ITO NW networks

The resistive random access memory (RRAM) has been attracting much attention because of its fast operation and high-density integration. The resistive switching (RS) is the basic unit of RRAM^46^. Sandwich structure is the most common RS with metal oxide (RS material) between two layers of metal electrode. The RS is characterized by the conversion of resistivity, which can be changed from a high-resistivity state (HRS) to a low-resistivity state (LRS) by applying a bias voltage and back from a reverse voltage.

**Figure 7:**
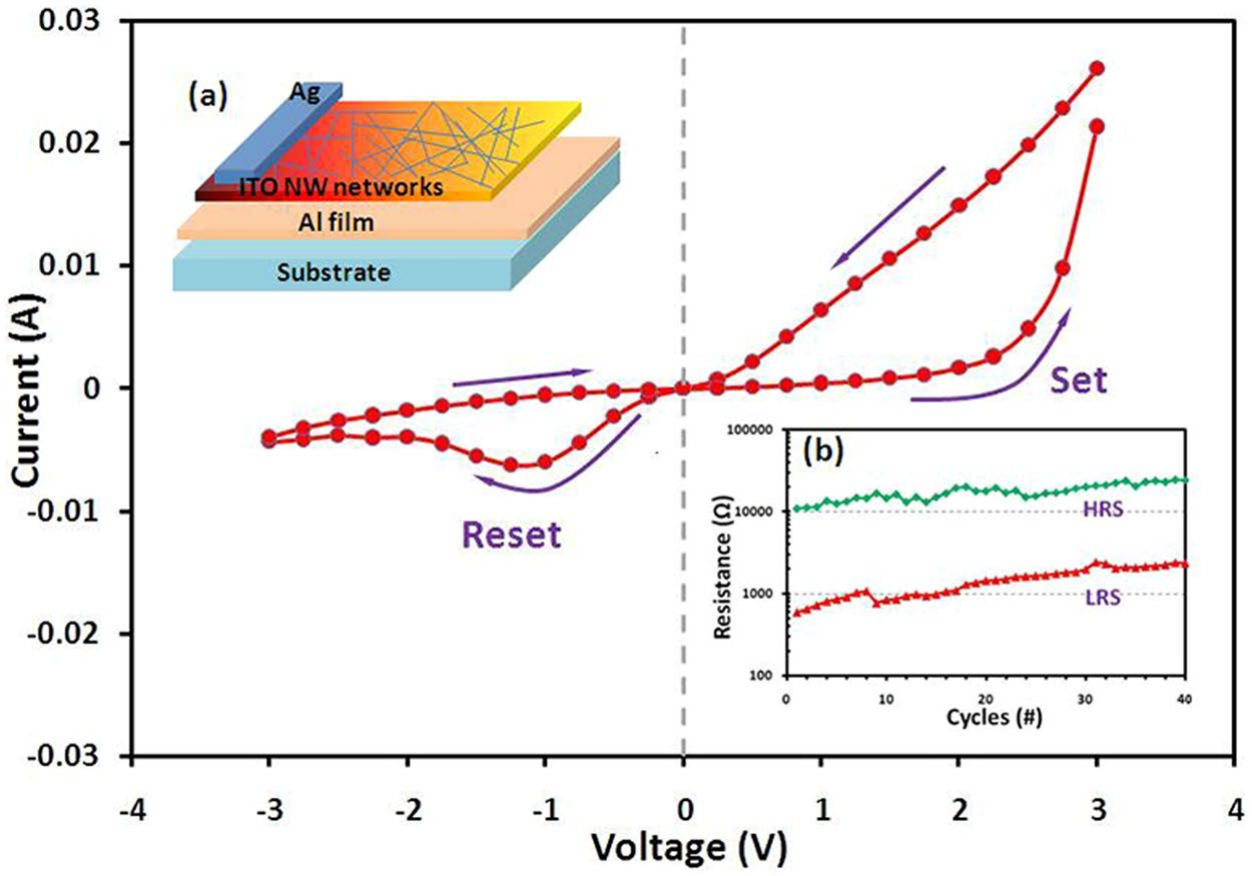
The typical I-V characteristics of Ag/ITO-NWs/Al capacitor. The inset image-(**a**) is the structural diagram of RS, and the inset image-(**b**) is the resistance evolution of HRS and LRS during the 100 resistive switching cycles. The arrows denote the sweeping direction of voltage.

The RS behavior of ITO-NW networks was first observed by using Ag/ITO-NWs/Al capacitor. The inset image-(a) of Fig. 7 shows the structure of resistive switching with ITO-NW networks. The aluminum with 500 nm was deposited as the bottom electrode on a sapphire substrate, and then the part of electrode leads was covered by high temperature adhesive tape. Next, the networks of interwoven ITO- NWs were fabricated by using PS spheres with size of 670 nm. Finally, we used silver conductive adhesive as the top electrode.

Figure 7 shows the typical I-V characteristics of sandwich-capacitor with ITO-NWs as RS material. This structure exhibits an asymmetric I-V relation with significant hysteresis. When the forward voltage is applied to a specific value (‘set’ voltage, V-set = 2.25 V), the leakage current becomes larger suddenly, indicating that the resistance of the capacitor switches to LRS from a HRS. This can be used as ‘on’ state. When the negative voltage is swept to a certain value (‘reset’ voltage, V-reset = −1.25 V), the current begins to reduce and the resistance goes back to HRS. This state can be marked as ‘off’ state. From the inset image-(b) of Fig. 7, the HRS and LRS of this device are (1.75 ± 0.39) × 10^4^ Ω and (1.46 ± 0.57) × 10^3^ Ω, respectively. The ratio of HRS/LRS is more than 10, which provided the possibility of high-density memory devices.

At present, the main two models for explaining the behaviors of RS in transition metal oxides are charge map model and conductive model^47^. The effect of oxygen vacancies on RS behaviors has been mentioned by both of the models. The existence of oxygen vacancies has a favorable effect on the RS behaviors. The ITO-NW networks are prepared under an anoxic atmosphere, and this means that there will be more oxygen vacancies in the synthesis process. High carrier density and low mobility of ITO NW networks were verified by Hall Effect measurement, so the electrical conductivity of nanowires could be limited under a certain voltage, corresponding with the HRS. When a particular voltage (V-set) was applied on the nanowires, the oxygen vacancies were accumulated at the Ag/ITO-NWs interface. The dimension of the ITO nanowire tip was sharp and the resistance was high due to the point contact, so the conductivity at tip was improved to form LRS. And then, the resistance switched back to the initial state (HRS) when the voltage is negatively swept to a special value (V-reset).

### ITO-NW networks applied on special morphological device

With the development of micro-nano technology, some special morphological devices have emerged, and how to further fabricate micro-nano structures on these special-shaped devices is a hot point. The PS-assisted ITO-NW networks are characteristics of facile efficient operation and free for different morphological substrates, so which can be applied as special transparent conductive layer.

**Figure 8:**
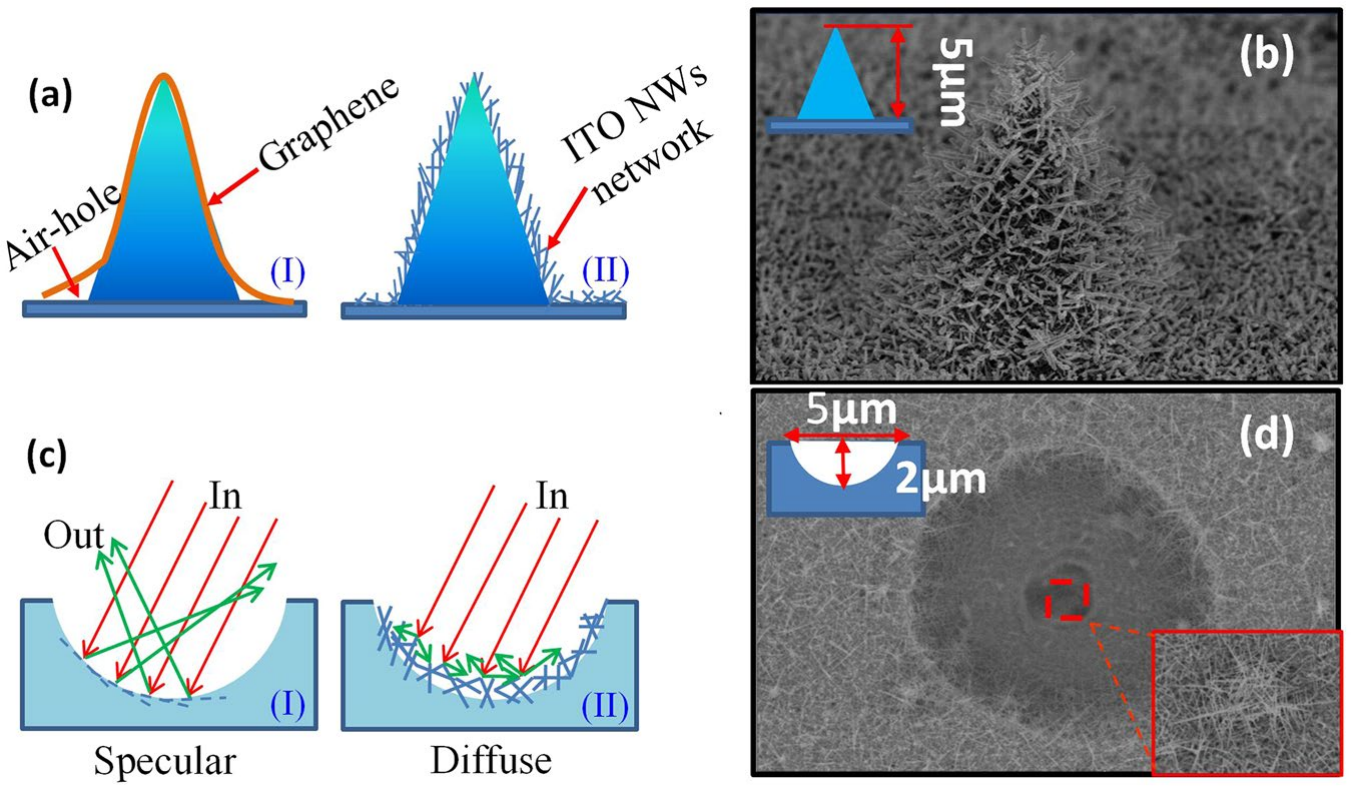
(**a**) The diagram of (i) graphene and (ii) ITO-NW networks on a single pyramid. (**b**) The SEM image of ITO-NW networks on pyramid. (**c**) The light reflection path in micropit (i) without and (ii) with ITO-NW networks. (**d**) The SEM image of ITO-NW networks in micropit.

The graphene was always used as a conductive electrode on the pyramid shaped nano-LED or flexible LED^48^, but a problem was often faced that there are some air holes between pyramid and substrate, as shown in Fig. 8a. The graphene has a certain mechanical strength and cannot fit with pyramids completely. The ITO-NW network prepared by PS-assisted method is a good solution to this problem. Figure 8b is the SEM image about a single pyramid with ITO-NW networks, and the surface of the pyramid was covered completely.

In order to improve the light collection capacity of solar cells, the wafer texturing of surface is always adopted. The ITO-NW networks can be used as a transparent conductive coarsened layer to further enhance the light collection capability. The principle is shown in Fig. 8c, when the light is cast into the micro-pits covered with ITO-NW networks, the original specular reflections become diffuse reflections, so that the light is almost confined in the micro-pits thus improving the utilization of light. The micro-pits (width ~5 *µ*m, depth ~2 *µ*m) were ablated on the surface of GaN substrate by the laser (λ = 355 nm, power = 0.04 W)^49^, then the ITO-NW networks were fabricated on that via PS spheres with diameter 500 nm. Figure 8d is the SEM of ITO-NW networks on a micropit. The whole micropit was covered by the ITO-NW networks, and the bottom of the micropit was completely covered.

It can be seen from the above, the PS-assisted ITO-NW networks can be realized on different morphological substrates, which greatly expanded the applications of ITO-NWs.

## Discussion

In conclusion, this work presents a method to fabricate ITO NWs via PS spheres by electron beam evaporation deposition. The growth mechanism of PS-assisted ITO NWs was analyzed in detail. The growth temperature is around 300 °C, and the PS can be removed easily and clearly. From XRD and HRTEM, the fabricated ITO NWs have a better crystallinity, and the interwoven NWs have the ability to form a well-conductive network. The morphology of ITO NWs can be regulated by the size of PS, and the large size PS spheres should be chosen to prepare the needle-shaped NWs.

The transmittance of ITO-NW networks layer is above 90% after 400 nm and the sheet resistance is ~200 Ω/□. When they applied on vertical blue and green LEDs, the light output power has been improved 31% and 26% at the work current, respectively. At the same time, the RS behavior of ITO-NWs was observed in Ag/ITO-NW networks/Al capacitor, and the ITO-NW networks can be used as RS material. The HRS/LRS is above 10, which is a clear memory window. This structure provides a potential for high-density RRAM.

At last, the application of ITO-NW networks on special morphological devices was discussed. Both of pyramids and micro-pits can be covered by ITO-NW networks completely. The networks can replace the graphene as conductive layer on some flexible devices, and as a transparent conductive coarsened layer to further enhance the light collection capability. So, the PS-assisted ITO-NW networks have potential attraction for scientific research and commercial development.

## Methods

### Self-Assembly of Polystyrene Spheres

The polystyrene spheres (PS, 5060 A, 10% solid, 15 ml) were purchased from Thermo Fisher Scientific. Firstly, a certain proportion (volume ratio = 1:3) of PS dispersion in alcoholic solution (AR, C_2_H_5_OH ≥ 99.8%) were prepared, and then the mixed solution was sonicated for 10 min. Next, the silicon wafer was used as a guide to transfer certain of dispersion solution to the surface of the deionized water. Thirdly, the hydrophilic treatment substrate was inserted into the water, and pulled slowly. Last, the samples were dried naturally in the air. Generally, in order to make the bonding more solid between substrate and PS film, the samples were needed to heat 10–15 min on heating plate at 100 °C.

### Growth of ITO nanowires

ITO source (In_2_O_3_:SnO_2_ = 90:10,wt%) was deposited on the template by EBE at a deposition rate of 0.1 nm/s for 15–20 min, with the chamber temperature stabilizing at 300 °C and pressure less than 5 × 10^−4^ Pa.

### Removal of Catalyst

The polystyrene (PS) material was removed by two ways: (i) according to the ‘like dissolves like’ principle, the chloroform (AR, CHCl_3_ ≥ 99.5%) was used to remove PS spheres. The ITO-deposited substrate was immersed in the chloroform solution for 20 min under ultrasonic condition. (ii) Using the property of PS evaporation at high temperature to remove catalyst, the sample was annealed at 300 °C for 5–10 min under the nitrogen condition.

### Structural Characterization

Bright-field transmission electron microscopy (TEM) analyses were conducted on a JEOL JEM 2100 F microscope (JEOL, Tokyo -Japan), operated at 200 kV acceleration voltage. The scanning electron microscopy (SEM) analyses were conducted on a HITACHI SU6600 Schottky Emission VP FE-SEM instrument. The X-ray diffraction (XRD) analyses were conducted on X’Pert PRO (Almelo-Netherlands). The X-ray Photoelectron Spectroscopy (XPS) analyses were carried out with a Kratos Axis Ultra DLD spectrometer using a monochromatic Al Kα source operated at 150 W. Spectra have been charge corrected to the main line of the C 1 s spectrum set to 284.3 eV.

## Electronic supplementary material

ITO nano-tree

## References

[1] Wan Q (2006). High- performance transparent conducting oxide nanowires. Nano Lett..

[2] Hill JJ, Banks N, Haller K, Orazem ME, Ziegler K (2011). An interfacial and bulk charge transport model for dye-sensitized solar cells based on photoanodes consisting of core–shell nanowires arrays. J. J. Am. Chem. Soc..

[3] Noh JH (2011). Nanowire-based three-dimensional transparent conducting oxide electrodes for extremely fast charge collection. Adv. Energ. Mater.

[4] Cairns DR (2000). Strain-dependent electrical resistance of tin-doped indium oxide on polymer substrates. Appl. Phys. Lett..

[5] Freeman AJ, Poeppelmeier KR, Mason TO, Chang RPH, Marks TJ (2000). Chemical and thin-film strategies for new transparent conducting oxides. MRS Bull..

[6] Buonsanti R, Llordes A, Aloni S, Helms BA, Milliron DJ (2011). Tunable infrared absorption and visible transparency of colloidal aluminum-doped zinc oxide nanocrystals. Nano Lett..

[7] Snure M, Tiwari A (2008). Band-gap engineering of Zn_1-x_GA_x_O nanopowders: synthesis, structural and optical characterizations. J. Appl. Phys..

[8] Bhosle V, Tiwari A, Narayan J (2006). Metallic conductivity and metal-semiconductor transition in Ga-doped ZnO. Appl. Phys. Lett..

[9] Bhosle V, Tiwari A, Narayan J (2006). Electrical properties of transparent and conducting Ga doped ZnO. J. Appl. Phys..

[10] Snure M, Tiwari A (2007). Structural, electrical, and optical characterizations of epitaxial Zn_1-x_GA_x_O films grown on sapphire (0001) substrate. J. Appl. Phys..

[11] Park KW (2013). Liquid crystal devices incorporating transparent Zn, Sn co-doped In_2_O_3_ electrodes prepared by direct inkjet-printing of nanosized particles. J. Phys. D.

[12] Kwak CH, Woo HS, Abdel-Hady F, Wazzan AA, Lee JH (2016). Vapor-phase growth of urchin-like Mg-doped ZnO nanowire networks and their application to highly sensitive and selective detection of ethanol. Sensor. Actuat. B-Chem.

[13] Wang Y (2015). Structure luminescence and photocatalytic activity of Mg-doped ZnO nanoparticles prepared by auto combustion method. Mat. Sci. Semicon. Proc.

[14] Mishra YK (2012). Crystal growth behaviour in Au-ZnO nanocomposite under different annealing environments and photoswitchability. J. Appl. Phys..

[15] Tiginyanu I (2016). Strong light scattering and broadband (UV to IR) photoabsorption in stretchable 3D hybrid architectures based on Aerographite decorated by ZnO nanocrystallites. Sci. Rep.

[16] Song J, Zeng H (2015). Transparent electrodes printed with nanocrystal inks for flexible smart devices. Angew. Chem. Int. Ed..

[17] Song J (2014). A general one-pot strategy for the synthesis of high-performance transparent-conducting-oxide nanocrystal inks for all-solution-processd devices. Angew. Chem. Int. Ed..

[18] Song J, Li J, Xu J, Zeng H (2014). Superstable transparent conductive Cu@Cu_4_Ni nanowire elastomer composites against oxidation, bending, stretching, and twisting for flexible and stretchable optoelectronics. Nano Lett..

[19] Paulowicz I (2015). Three-dimensional SnO_2_ nanowire networks for multifunctional applications: from high-temperature stretchable ceramics to ultra-responsive sensors. Adv. Electron. Mater.

[20] Lupan O (2015). Rapid switching and ultra-responsive nanosensors based on individual shell-core Ga_2_O_3_/GaN:Ox@SnO_2_ nanobelt with nanocrystalline shell in mixed phases. Sensor. Actuat. B-Chem.

[21] Joanni E (2007). Dye-sensitized solar cell architecture based on indium–tin oxide nanowires coated with titanium dioxide. Scr. Mater.

[22] Wang GJ, Chen HT, Yang H (2008). Fabrication of Crystalline Indium Tin Oxide Nanobasket Electrodes using Aluminum Anodic Oxide Template. Jpn. J. Appl. Phys..

[23] Yang F, Forrest SR (2008). Photocurrent Generation in Nanostructured Organic Solar Cells. ACS Nano.

[24] Yu P (2010). Embedded indium-tin-oxide nanoelectrodes for efficiency and lifetime enhancement of polymer-based solar cells. Appl. Phys. Lett..

[25] Fung MK (2011). Indium tin oxide nanorod electrodes for polymer photovoltaics. ACS Appl. Mater. Interfaces.

[26] Rider DA (2011). Indium tin oxide nanopillar electrodes in polymer/fullerene solar cells. Nanotech.

[27] Savu R, Joanni E (2008). Effect of processing conditions on the nucleation and growth of indium-tin-oxide nanowires made by pulsed laser ablation. J. Mater. Sci..

[28] Chang WC, Kuo CH, Lee PJ, Chueh YL, Lin SJ (2012). Synthesis of single crystal Sn-doped In_2_O_3_ nanowires: size-dependent conductive characteristics. Phys. Chem.

[29] Park HK, Yoon SW, Chung WW, Min BK, Do YR (2013). Fabrication and characterization of large-scale multifunctional transparent ITO nanorod film. J. Mate. Chem. A.

[30] Dattoli EN, Lu W (2011). ITO nanowires and nanoparticles for transparent films. MRS Bull..

[31] Johnson MC, Aloni S, McCready DE, Bourret-Courchesne ED (2006). Controlled vapor-liquid-solid growth of indium, gallium, and tin oxide nanowires via chemical vapor transport. Cryst. Growth Des..

[32] Meng G (2013). Impact of preferential indium nucleation on electrical conductivity of vapor–liquid–solid grown indium–tin oxide nanowires. J. Am. Chem. Soc..

[33] Wan, Q., Sun, J. & Liu, H. “*Nanowires-Implementations and Applications*” ISBN:978-953-307-318-7, InTech, Chapter 4 59–98, July (2011).

[34] Beaudry AL, Tucher RT, Laforge JM, Taschuk MT, Brete MJ (2012). Indium tin oxide nanowhisker morphology control by vapour–liquid–solid glancing angle deposition. Nanotech.

[35] Kunar RR, Rao KN, Rajanna K, Phani AR (2014). Low temperature and self catalytic growth of ultrafine ITO nanowires by electron beam evaporation method and their optical and electrical properties. Mater. Res. Bull..

[36] Yamamoto N, Morisawa K, Murakami J, Nakatani Y (2014). Formation of ITO nanowires using conventional magnetron sputtering. ESC Solid State Lett.

[37] Qiang L (2016). Electro-optical properties of low temperature growth insium-tin-oxide nanowires using polystyrene spheres as catalyst. Nanoscal Res. Lett.

[38] Wan N (2010). Broadband anti-reflection and enhanced field emission from catalyst-free grown small-sized ITO nanowires at a low temperature. Acta Mater..

[39] Garnett E, Yang P (2010). Light Trapping in Silicon Nanowire Solar Cells. Nano Lett..

[40] Sivakov V (2009). Silicon nanowire-based solar cells on glass: synthesis, optical properties, and cell parameters. Nano Lett..

[41] Li L (2015). Controlled synthesis of tin-doped indium oxide (ITO) nanowires. J Cryst. Growth.

[42] Kunar RR, Gaddam V, Rao KN, Rajanna K (2014). Low temperature VLS growth of ITO nanowires by electron beam evaporation method. Mater. Res. Express.

[43] Tsai MA, Wang HW, Yu P, Kuo HC, Lin SH (2011). High extraction efficiency of GaN-based vertical-injection light-emitting diodes using distinctive indium-tin-oxide nanorod by glancing-angle deposition. Jpn. J. Appl. Phys..

[44] Leem YC (2014). Enhanced optical output power of InGaN/GaN vertical light-emitting diodes by ZnO nanorods on plasma-treated N-face GaN. Nanoscal.

[45] Huh C (2014). Enhancement in light emission and electrical efficiencies of a silicon nanocrystal light-emitting diode by indium tin oxide nanowires. Appl. Phys. Lett..

[46] Park J, Lee S, Yong K (2012). Photo-stimulated resistive switching of ZnO nanorods. Nanotech.

[47] Sawa A (2008). Resistive switching in transition metal oxides. Mater. Today.

[48] Wang L (2014). *In situ* fabrication of bendable microscale hexagonal pyramids array vertical light emitting diodes with graphene as stretchable electrical interconnects. ACS Photonics.

[49] Wang S (2016). Laser patterning of Y_3_Al_5_O_12_:Ce^3+^ ceramic phosphor platelets for enhanced forward light extraction and angular color uniformity of white LEDs. Opt. Express.

