# Supplementary material for: Fabrication and application of indium-tin-oxide nanowire networks by polystyrene-assisted growth
Source: Sci Rep. 2017 May 9;7:1600. doi: 10.1038/s41598-017-01385-0 (PMC5431639; doi:10.1038/s41598-017-01385-0)
Supplement: Supplementary file 1 — ITO nano-tree [file 41598_2017_1385_MOESM1_ESM.doc]

**Fabrication and application of indium-tin-oxide nanowire networks by polystyrene-assisted growth**

Qiang Li^1,2^, Feng Yun^1,2*^, Yufeng Li^1,2^, Wen Ding^1^, and Ye Zhang^2^

^1^Key Laboratory of Physical Electronics and Devices for Ministry of Education and Shaanxi Provincial Key Laboratory of Photonics & Information Technology, Xi’an Jiaotong University, Xi’an, Shaanxi, P. R. China

^2^Solid-State Lighting Engineering Research Center, Xi’an Jiaotong University, Xi’an, Shaanxi, P. R. China

*fyun2010@mail.xjtu.edu.cn


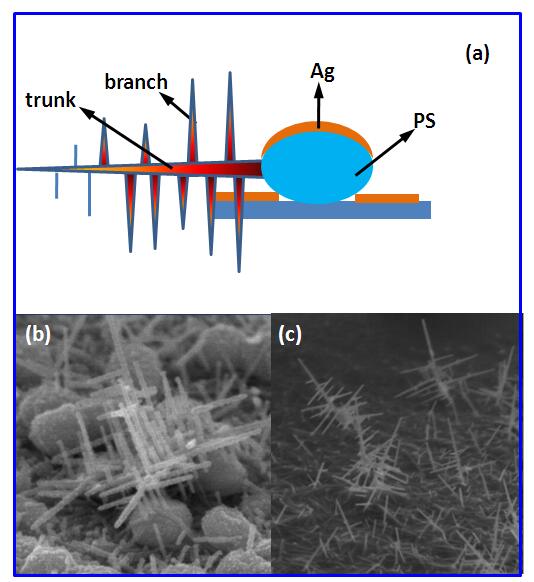


**Fig.S1** (a) The preparation diagram of ITO nano-tree. The SEM images of (b) nano-tree on PS, and (c) nano-tree on substrate.

The nano-tree is the growth morphology of single ITO nanowire with trunk and branch, which can be prepared directly under the condition of sufficient PS. Firstly, the PS spheres with diameter of 500 nm (or bigger) were prepared on substrate, and then the silver with thickness of 80 nm was deposited to cover part of PS. The purpose is to guide the growth direction of ITO nanowires, because the nanowires can only grow along the uncapped part of PS, as shown in Fig.S1(a). The Fig.S1(b) shows the SEM image of nano-tree grown on PS sphere. All the branches are perpendicular to the trunk. In addition, if we do not use the directional guidance method, we can also find the existence of ITO nano-tree in the growth region of sufficient PS on some samples, when the ITO-NW networks were prepared via PS, like as Fig.S1(c).
